# Upskilling in Healthy Longevity Medicine and Its Association With Physicians’ Implementation Intent and Self-Reported Clinical Confidence: Cross-Sectional Observational Study

**DOI:** 10.2196/83779

**Published:** 2026-03-19

**Authors:** Evelyne Bischof, Dominika Wilczok, James L Kirkland, Bhirau Wilaksono, Christine Yuan Huang, Suwanna Suwannaphong, Wanviput Sanphasitvong, Dalila Čamdžić, Carolina Hernandez, Yoko Madea, Hidekazu Yamada, Melissa Alexandre Fernandes, Ricardo Gaminha Pacheco, Fabiano M Serfaty, Fernanda Calvo-Fortes, Amit Goldman, Andrea B Maier, Alexey Moskalev, Morten Scheibye-Knudsen, Alex Zhavoronkov

**Affiliations:** 1 Sheba Longevity Center, Sheba Medical Center, Gray Faculty of Medical & Health Sciences, Tel Aviv University, Sheba Road 2, Ramat Gan, 5265601, Israel, 86 15000864674; 2 Shanghai University of Medicine and Health Sciences, Shanghai, China; 3 Duke University, Durham, NC, United States; 4 Duke Kunshan University, Kunshan, Jiangsu, China; 5 Center for Advanced Gerotherapeutics, Division of Endocrinology and Metabolism, Department of Medicine, Cedars Sinai Health Sciences University, Los Angeles, CA, United States; 6 Center for Healthy Longevity, Faculty of Medicine, Universitas Negeri Makassar, Indonesia; 7 Department of Biochemistry, Faculty of Medicine, Universitas Negeri Makassar, Indonesia; 8 Quantum Life Inc, Hong Kong, China (Hong Kong); 9 VitalLife, Bumrungrad International Hospital, Bangkok, Thailand; 10 Sarajevo School of Science and Technology, Sarajevo, Bosnia and Herzegovina; 11 Departent of Biomedicine, University of Barcelona, Barcelona, Spain; 12 Madea Clinic, Okayama, Japan; 13 Anti-Aging Center, Kindai University, Osaka, Japan; 14 Novartis Institutes for BioMedical Research, Basel, Switzerland; 15 Insilico Medicine Hong Kong Ltd, Hong Kong, China (Hong Kong); 16 Departent of Biomedicine, Universidade do Estado do Rio de Janeiro, Rio de Janeiro, Brazil; 17 School of Nursing, Federal University of Minas Gerais, Belo Horizonte, Brazil; 18 Saïd Business School, University of Oxford, Oxford, United Kingdom; 19 Healthy Longevity Translational Research Program, Yong Loo Lin School of Medicine, National University of Singapore, Singapore; 20 NUS Academy for Healthy Longevity, Yong Loo Lin School of Medicine, National University of Singapore, Singapore; 21 Department of Human Movement Sciences, Faculty of Behavioral and Movement Sciences, Amsterdam Movement Sciences, Vrije Universiteit, Amsterdam, The Netherlands; 22 Petrovsky Russian Research Center for Surgery, Moscow, Russian Federation; 23 Center for Healthy Aging, Department of Cellular and Molecular Medicine, University of Copenhagen, Copenhagen, Denmark; 24 Insilico Medicine US Inc, Cambridge, MA, United States

**Keywords:** longevity, survey, continuing medical education, clinical training, professional development

## Abstract

**Background:**

Structured educational programs for physicians in healthy longevity medicine (HLM) remain scarce. No published data yet document the impact of longevity-focused medical education on physicians. This study assesses the ramifications of the HLM curriculum, certified by the American Council for Continuing Medical Education, on physicians’ confidence in their knowledge of HLM and clinical practice.

**Objective:**

This study aimed to evaluate the impact of accredited HLM education on physicians’ confidence in knowledge and practice patterns, examining self-reported integration of HLM principles, professional attitudes, and career trajectories to determine the translational value of structured curricula in the emerging medical discipline.

**Methods:**

A cross-sectional online survey was conducted between March and April 2024 among physicians who had completed accredited HLM courses between January 2023 and February 2024. Invitations were sent globally to 590 eligible physicians; trainees and students were excluded. A total of 113 (19.2%) respondents completed the survey and were included in the analysis. The survey assessed self-reported changes in clinical implementation, confidence in HLM-related knowledge, and professional attitudes following course completion. Descriptive statistics and logistic regression analyses were performed (*P*<.05).

**Results:**

Respondents represented 42 nationalities and were primarily trained in family medicine (n=31, 27.4%) and internal medicine (n=18, 15.9%). Overall, 96.5% (n=99) of the respondents reported increased confidence in HLM-related knowledge, with 47.8% (n=55) indicating substantial improvement. More than half of the respondents (n=63, 55.8%) reported integrating HLM principles into routine patient assessments, and 80.5% (n=91) of the respondents reported more frequent discussions related to health span–focused care. In addition, 23% (n=26) of the respondents initiated aging biomarker testing, 48.7% (n=55) increased the testing frequency, 52.2% (n=59) reported a shift in their perspective on aging, and 73.5% (n=83) anticipated full integration of HLM into mainstream medicine. Physicians practicing in specialized care demonstrated higher odds of reporting increased confidence in HLM knowledge compared with those in primary and preventive care (odds ratio 4.46, 95% CI 1.55‐12.79; *P*=.005).

**Conclusions:**

Accredited education in HLM is associated with enhanced confidence in HLM knowledge, increased clinical engagement with HLM practices, and a shift in aging-related care paradigms. These findings underscore the critical role of structured HLM curricula in bridging the translational gap between geroscience and everyday medical practice. Nevertheless, systemic health care barriers impede widespread implementation, warranting policy-level strategies to support health span–oriented education and care models.

## Introduction

As populations age globally, there is an increasing need for rigorous, evidence-based education to prepare health care professionals to manage the complex, multimorbidity-driven needs of older adults. Healthy longevity medicine (HLM) represents an emerging clinical framework aimed at optimizing health span through proactive, evidence-based diagnostics and interventions grounded in geroscience—the discipline that studies the biological mechanisms of aging; their role in age-related chronic diseases; and the development of interventions to delay, prevent, or reverse these processes [1]. While geroscience has advanced rapidly over the past decade, translating these breakthroughs into clinical practice remains limited by a lack of data from prospective, well-designed clinical trials; formalized education; clinical practice protocols; and integration into existing health care systems [2]. Bridging this translational gap is essential to ensure that the benefits of geroscience reach patients in a timely, equitable, and scalable manner. Parallel to these scientific advances, new diagnostic and therapeutic approaches relevant to HLM are emerging, including dual-purpose interventions that target fundamental aging mechanisms while addressing disease-specific outcomes [3], as well as biological aging clocks, increasingly incorporating multimodal and artificial intelligence–based methods [4]. These developments expand the potential scope of care but also introduce complexity that exceeds conventional, disease-based training models. As a result, physicians require dedicated education not only to apply these tools responsibly in clinical settings but also to contribute meaningfully to clinical trial design, interpretation, and translational research efforts. At the same time, the rapid expansion of the “healthy longevity” field has been accompanied by a proliferation of nonstandardized, commercially driven, and sometimes misleading information, which can blur the distinction between evidence-based preventive medicine and unvalidated interventions. Therefore, structured educational initiatives grounded in established geriatric, preventive, and translational aging science may play a critical role in equipping clinicians with the skills needed to critically appraise emerging evidence, apply validated interventions appropriately, and safeguard patients against misinformation. Strengthening evidence-based training in this domain is essential not only for clinical quality and patient safety but also for maintaining professional and public trust as the field continues to evolve.

Despite this need, formal medical education in HLM remains scarce. Since 2020, the Longevity Education Hub has provided the first and only American Council for Continuing Medical Education–accredited educational framework designed specifically for practicing physicians and health care professionals. The courses represent a systematic attempt to equip physicians with the tools necessary to apply knowledge of the biology of aging in a clinical setting. Importantly, this curriculum has been adopted into the required coursework of medical schools within 2 major universities (State University of Massakar in Indonesia and King Mongkut’s Institute of Technology Ladkrabang in Thailand), marking a historic integration of HLM into formal medical education.

To expand physician education beyond the core continuing medical education (CME) courses, the first conference event dedicated to HLM, Longevity Medicine Day, was held in 2021 at the Aging Research and Drug Discovery Conference. This event became an annual feature and was expanded into the Healthy Longevity Medicine Track by 2024 [5,6]. In response to the growing interest in HLM among clinicians, the Healthy Longevity Medicine Society was established in 2022, providing a structured professional body to support clinicians and standardize practices within the field [7]. These milestones provided a theoretical and educational foundation for integrating HLM into clinical settings. Parallel to these scientific developments, institutional recognition of HLM has grown. In 2024, the Department of Health Abu Dhabi became the first regulatory body to officially recognize HLM protocols and initiate their integration into clinical settings [8].

Nevertheless, empirical data evaluating how such educational initiatives influence physicians’ knowledge, attitudes, and clinical implementation remain limited. This gap is particularly consequential as HLM gains visibility in the consumer health sector, where the absence of validated clinical standards increases the risk of unproven interventions and commercial exploitation. Previous experience in other emerging or high-risk domains, such as pain management, demonstrates that mandatory and structured physician education can improve clinical confidence and promote safer practice patterns; for example, California’s CME mandate in pain management was associated with self-reported positive practice changes in up to 90% of physicians at follow-up [9]. More broadly, CME-based interventions have been shown to enhance physician knowledge, confidence, and selected patient outcomes across medical disciplines [10,11].

Understanding this dynamic is crucial for guiding the next phase of curriculum development, policy adoption, and the broader institutionalization of HLM. In addition, any educational effort, particularly in emerging medical fields, requires systematic quality control to ensure its effectiveness and scalability. To meet this need, we conducted a cross-sectional study to evaluate physicians who have undergone such formal education in HLM. This study presents, to the best of our knowledge, the first global survey of physicians who have completed the HLM curriculum, evaluating its perceived impact, clinical integration, and barriers that remain. In doing so, we offer empirical insights into how frontline clinicians are shaping the transformation of geroscience into a defined, evidence-based clinical discipline.

## Methods

### Ethical Considerations

This study involved an anonymous, voluntary online survey of physicians who had completed accredited HLM courses.

In accordance with institutional and local policies, formal ethics board approval was not sought, as the study did not constitute human participant research requiring review. Institutional review board approval was not sought, as the study was designed and conducted as an anonymous, voluntary survey of physicians that collected no patient data, no sensitive personal information, and no identifiable data and posed no risk to participants. We followed the EC Horizon and the US Department of Health and Human Services (45 CFR 46.104(d)(2)) regulation [12].

No personal identifiers were collected. Informed consent was implied by participants’ decision to complete the survey, and no financial or material compensation was provided. This study was conducted in accordance with the STROBE (Strengthening the Reporting of Observational Studies in Epidemiology; Checklist 1) guidelines for cross-sectional studies.

### Study Design

This cross-sectional observational study was conducted between March and April 2024. Physicians who completed the Longevity Education Hub’s online Longevity Medicine 101 and/or Longevity Medicine 201 courses self-administered and remotely completed the anonymous online survey assessing changes in clinical practice, confidence in HLM knowledge, and perspectives on HLM.

### Participants

**Figure 1.**
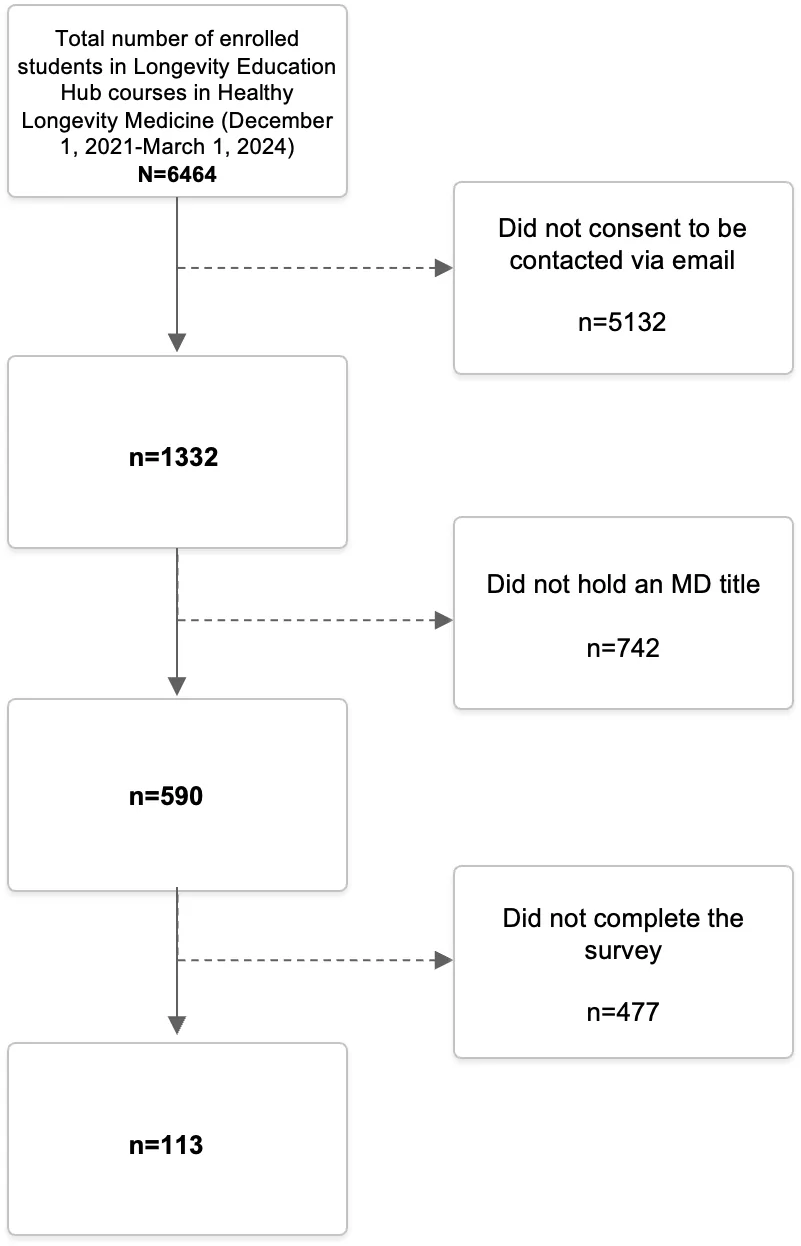
Flowchart for participant recruitment. MD: Doctor of Medicine.

The study population was derived from all individuals who enrolled in the Longevity Medicine 101 and 102 courses offered through the Longevity Education Hub on Teachable between December 1, 2021, and March 1, 2024 (N=6464). Participants were excluded if they did not complete all course modules or did not pass the final quiz (n=3241, 50.1%), leaving 3223 (49.9%) course completers. Among them, those who did not report holding a Doctor of Medicine title upon course enrollment were excluded (n=1561, 24.1%), resulting in 1662 (25.7%) physicians. Junior physicians in training or medical students were further excluded (n=1072, 16.6%) because HLM is an emerging discipline without direct pathways to board-certified specialization; therefore, entry into HLM practice typically requires substantial clinical experience in one’s primary discipline—an experience that trainees, by definition, lack. Therefore, trainees were excluded. The study survey was sent to all remaining 590 (9.1%) qualifying practicing physicians. Figure 1 illustrates the process of selecting the participants. The participants did not receive any monetary compensation for their responses.

Demographic data, including nationality and medical specialization, were collected. For analytical purposes, medical specialties indicated by the participants were grouped according to their primary role within the health care delivery continuum. Primary and preventive care and general practice (PPG) encompass specialties characterized by first-contact patient care, longitudinal responsibility, and a broad scope of practice, with a strong emphasis on prevention, risk stratification, and comprehensive health management. This group included general practice, family medicine, and related primary care disciplines, as displayed in Table 1. In contrast, specialized care and advanced procedures (SPEC) comprised specialties predominantly engaged through referral, with a more focused scope of practice centered on specific organ systems, disease categories, or procedural interventions.

**Table 1.** Geographic distribution (derived from nationality), medical specialization, and professional status of survey respondents (N=6464 and n=113).

Item	Values, n (%)
Region (derived from nationality)	
Asia	24 (21.2)
Australia and Oceania	6 (5.3)
Africa	2 (1.8)
Europe	45 (39.8)
South America	11 (9.7)
North America	17 (15)
Middle East	8 (7)
Primary and preventive care and general practice	
Family medicine	31 (27.4)
Internal medicine	18 (15.9)
Public health	3 (2.6)
Integrative medicine	2 (1.8)
Community medicine	1 (0.9)
Functional medicine	1 (0.9)
Precision preventive medicine	7 (6.2)
Specialized care and advanced procedures	
Oncology	5 (4.4)
Orthopedics	3 (2.6)
Dermatology	3 (2.6)
Lifestyle medicine	3 (2.6)
Pain management	3 (2.6)
Endocrinology	3 (2.6)
Cardiology	3 (2.6)
Sports medicine	2 (1.8)
Clinical nutrition	1 (0.9)
Neuropsychiatry	1 (0.9)
Obstetrics and gynecology	1 (0.9)
Hematology	2 (1.8)
Regenerative medicine	1 (0.9)
Plastic surgery	1 (0.9)
Neurology	3 (2.6)
Urology	1 (0.9)
Psychiatry	2 (1.8)
Psycho-neuro-endocrino-immunology	1 (0.9)
MD^a^+MBA^b^ in hospital administration	1 (0.9)
Medical genetics	3 (2.6)
Neurosurgery	1 (0.9)
Physical medicine and rehabilitation	1 (0.9)
General surgery	4 (3.5)
Transplantology	1 (0.9)
Practice status	
Practicing MD (public)	34 (30.1)
Practicing MD (private)	61 (54)
Practicing MD (<40% clinical time)	7 (6.2)
Not practicing MD	11 (9.7)

a MD: Doctor of Medicine.

b MBA: Master of Business Administration.

### Intervention

Longevity Education Hub’s Longevity Medicine 101 and Longevity Medicine 201 courses provide structured, free-of-charge, accredited education in HLM for physicians. The courses are delivered in an online, asynchronous format with a total instructional duration of approximately 2 hours and 40 minutes and conclude with a final assessment requiring a minimum score of 80% for certification. The curriculum includes an introduction to HLM and core definitions; the epidemiology of aging; the scientific basis of aging from biogerontology to clinical translation; definitions and biological mechanisms of aging; therapeutic interventions and innovations in HLM, including geroprotectors and psychological aspects of aging; methods for measuring aging, including biological aging, biomarkers of aging, biochronology, and aging clocks; and the clinical application of HLM, encompassing diagnostic frameworks, structured clinical protocols, and integration of HLM into clinical practice. Emerging applications of artificial intelligence in longevity research and clinical workflows are also introduced.

### Assessment

A 20-item survey instrument was developed using a structured 3-phase process. First, initial items were generated based on a comprehensive literature review and consultation with subject-matter experts in the field of HLM. This was followed by content validation conducted by a panel of 4 independent experts in medical education and HLM, ensuring the relevance and clarity of the survey items. Finally, the instrument was pilot-tested with a sample of 20 physicians, although responses from the pilot were not included in the final analysis. The finalized survey demonstrated high internal consistency, with a Cronbach α of 0.72. The mandatory questions took up to 10 minutes to complete. The primary outcomes of this study were self-reported confidence in HLM knowledge after the training, measured using a Likert scale, and the integration of HLM principles into clinical assessments, assessed through frequency-based response options. Secondary outcomes included the frequency of initiating longevity-related discussions with patients, the adoption and frequency of aging biomarker testing, the perceived efficacy of HLM education in addressing demographic challenges, shifts in perspectives on aging and longevity, and anticipated growth of HLM as a medical field. The survey also assessed participants’ interest in further HLM education, their support for the accreditation of HLM as a specialty, and their willingness to join an HLM consortium. The last question was nonmandatory and open-ended, inviting participants to provide any additional comments.

The survey was distributed via email with a link to a Google Forms questionnaire. The distribution consisted of 2 send-outs spaced 2 weeks apart to maximize response rates. Two weeks after the initial invitation, all recipients received an automatic reminder, regardless of whether they had already completed the survey. To prevent multiple submissions, each participant was permitted to complete the survey only once.

### Statistical Analysis

Statistical analyses were conducted in R (version 4.2.0; R Development Core Team Foundation for Statistical Computing). Data quality checks confirmed valid ranges and internal consistency of responses. Because the survey platform (Google Forms) required completion of all essential items before submission, there were no missing values for mandatory variables, and no imputation or casewise exclusion was performed. Descriptive statistics (counts and percentages) summarized demographics and survey responses overall and by group. Between-group differences (SPEC vs PPG) in course-related outcomes were assessed using separate logistic regression models (1 model per outcome). The group was included as the primary independent variable, with PPG serving as the reference category. Model results were reported as odds ratios (ORs), 95% CIs, and *P* values. Statistical significance was set at *P*<.05.

## Results

The survey was sent to 590 eligible physicians, of whom 113 (19.2%) completed the survey (Figure 1). Table 1 summarizes the demographic and professional characteristics of the respondents, including nationality, medical specialty, and practice setting. The sample included physicians of 42 nationalities. Regional groupings follow standard continental divisions, with the Middle East presented separately as a commonly recognized geopolitical region in international health research. Regionally (based on nationality), the largest proportions of physicians were European (n=45, 39.8%), Asian (n=24, 21.2%), North American (n=17, 15%), and Middle Eastern (n=9, 8%). The most prevalent specialties were family medicine (n=31, 27.4%) and internal medicine (n=18, 25.6%). Regarding practice setting, 54% (n=61) of the respondents worked in private practice and 30.1% (n=34) in public settings.

**Figure 2.**
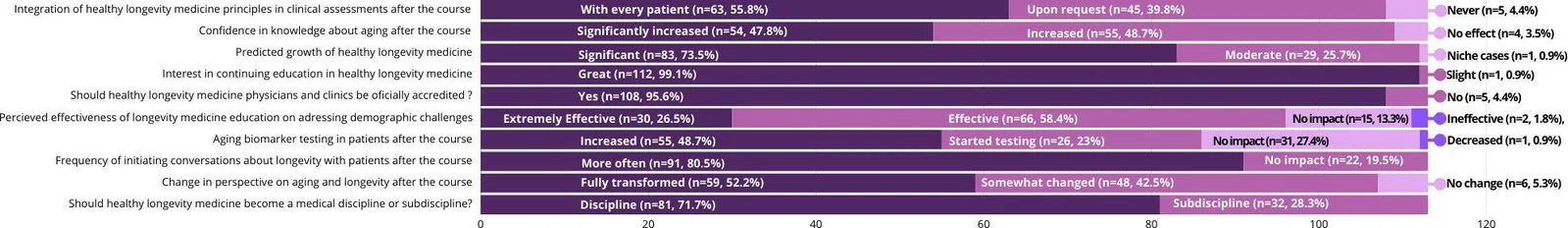
Impact of accredited healthy longevity medicine (HLM) education.

Regarding the primary outcomes, the analysis of the responses indicated positive feedback on the impact of the course on participants’ confidence in their knowledge of HLM. A total of 99 (96.5%) of the 113 respondents reported an increase in confidence in their knowledge, with 54 (47.8%) respondents noting a significant improvement, as shown in Figure 2. In terms of clinical integration, 63 (55.8%) participants reported implementing HLM principles into routine assessments for all patients, with 45 (39.8%) participants applying these principles on special request (Figure 2).

Regarding secondary outcomes, 59 (52.2%) respondents indicated that the courses fully transformed their perspective on aging and healthy longevity (Figure 2). In addition, 26 (23%) participants who had not previously engaged in aging biomarker testing began incorporating it into their practice, while 55 (48.7%) respondents increased the frequency of such tests (Figure 2). Most of the participants (84.9%) perceived HLM education as an effective or extremely effective means of addressing demographic challenges (Figure 2). Furthermore, 91 (80.5%) respondents reported engaging in more frequent discussions about healthy longevity with their patients after completing the course (Figure 2). Looking ahead, 83 (73.5%) participants expected HLM to become fully integrated into mainstream medicine, while 29 (25.7%) participants believed it would remain in specialized clinics (Figure 2). The findings also show strong support for the formal accreditation of physicians and clinics specializing in HLM, with 108 (95.6%) respondents in favor (Figure 2). Most of the participants (n=112, 99.1%) participants expressed interest in further HLM education (Figure 2). Finally, 71.7% (n=81) of respondents believed that HLM will eventually be recognized as an official medical discipline (Figure 2).

Physicians reported various career impacts following HLM education. Most respondents (n=39, 34.5%) reported joining a group of HLM physicians. A notable proportion of respondents (n=36, 31.9%) indicated that the education did not result in significant changes to their medical career. In addition, 24 (21.2%) respondents reported opening a new HLM clinic, 4 (3.5%) joined a pre-existing HLM clinic, and 10 (8.9%) participants did both.

**Figure 3.**
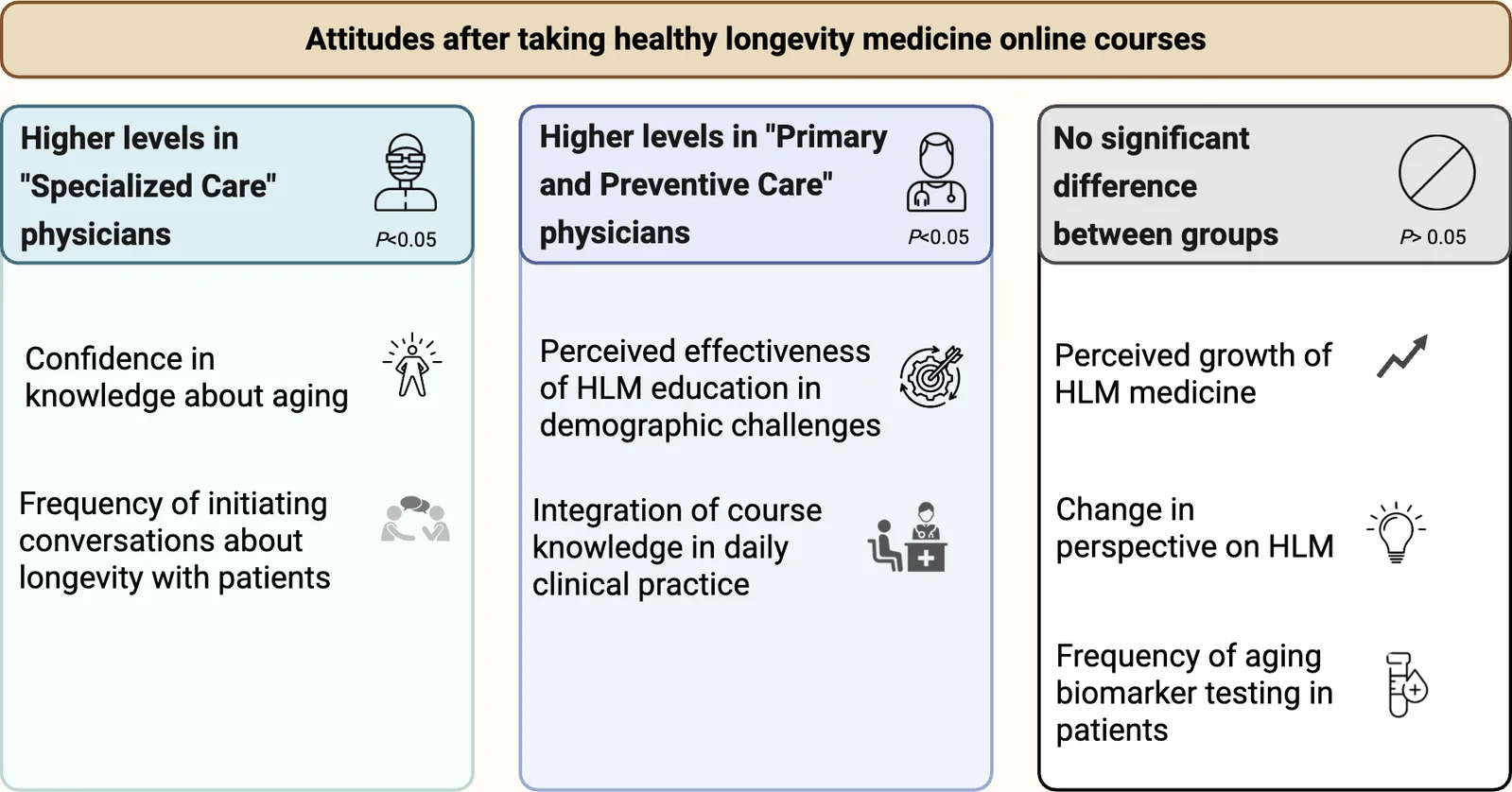
Differences in effects of healthy longevity medicine (HLM) education between primary and preventative care physicians and specialized care physicians.

Logistic regression analysis revealed differences between groups’ key educational takeaways, as illustrated in Figure 3. Confidence gain in HLM knowledge was significant in the SPEC group (OR 4.46, 95% CI 1.55‐12.79; *P*=.005), with SPEC physicians having almost 4.5 times higher odds of reporting increased confidence after HLM education than PPG physicians. A higher frequency of HLM-related discussions was associated with a nearly threefold greater likelihood of occurring in the SPEC group (OR 2.98, 95% CI 1.30‐6.83; *P*=.01). In contrast, the PPG physicians reported a greater perceived effectiveness of HLM education (OR 0.29, 95% CI 0.12‐0.75; *P*=.01). The PPG group also had a greater integration of healthy longevity principles into clinical assessments (OR 0.70, 95% CI 0.53‐0.91; *P*=.009).

Although none of the additional variables reached statistical significance, several trends emerged. Physicians in the SPEC group were more likely to report a more positive change in perspective on aging and healthy longevity after completing the courses (OR=0.389, 95% CI 0.13‐1.14; *P*=.09), as well as to predict greater growth of HLM (OR 0.73, 95% CI 0.26‐2.04; *P*=.55). However, PPG physicians reported more frequent testing of biomarkers relevant to aging processes (OR 1.49, 95% CI 0.71‐3.14; *P*=.29).

The open-ended question regarding any additional comments the participants could have about HLM revealed multiple systemic barriers that continue to hinder its integration into clinical practice. The participants’ answers revealed them to be financial disincentives embedded in fee-for-service models that disfavor preventive, time-intensive care; regional disparities in infrastructure and adoption of preventive frameworks; and the lack of standardized clinical guidelines translating scientific advances into routine medical workflows. The participants noted that the absence of formal regulatory recognition, limited accreditation pathways, and uneven diagnostic access further compound the challenge.

## Discussion

### Principal Findings

This study found that accredited, online HLM courses are associated with significant improvements in physician-reported confidence in the knowledge of HLM and increased clinical integration of healthy longevity–focused practices.

More than half of the participants (n=63, 55.8%) reported routinely integrating HLM principles into assessments for all patients, while the rest applied these principles selectively, suggesting that educational gains translated into context-dependent, clinical behaviors. Notably, nearly all participants (n=109, 96.5%) reported increased confidence in HLM-related knowledge, with about half (n=54, 47.8%) reporting substantial improvement. Physicians in the SPEC group were 4 times more likely to report increased confidence than those in the PPG group. This difference may reflect how the course content aligned with routine clinical approaches across practice settings. Because several HLM concepts overlap with preventive and systems-based care commonly used in primary care, some PPG physicians may have perceived smaller relative gains in knowledge confidence despite completing the same course. The observed differences between SPEC and PPG physicians suggest that tailored educational approaches may be necessary to address the unique needs of diverse clinical sectors. The results support the hypothesis that structured online education can enhance physician engagement with HLM, suggesting a potential role for scalable digital learning in bridging knowledge gaps in HLM.

Beyond the primary outcomes, the findings indicate wider shifts following HLM education. More than half of the respondents (n=59, 55.2%) reported a complete transformation in their perspective on aging and healthy longevity, and the majority (n=96, 84.9%) perceived HLM education as an effective or extremely effective approach to addressing demographic challenges. Notably, 80.5% (n=91) of all physicians reported an increase in patient-facing healthy longevity conversations postcourse, indicating a meaningful shift in engagement strategies. Moreover, a general increase in biomarker use observed in both groups (n=55, 48.7% of all respondents increased their use) aligns with a broader movement within the HLM field toward earlier risk stratification and individualized preventive strategies. However, there is increasing recognition that biomarkers of aging are currently more robust as research and monitoring tools than as definitive clinical decision instruments [13-15]. Recent consensus efforts emphasize their value for standardizing outcomes in intervention studies and highlight the necessity of using a variety of biomarkers for accurate reflection of biological aging [16]. Despite the strong educational uptake, belief in the future growth of HLM and changes in perspectives on aging did not reach statistical significance in the regression model. Critically, nearly all respondents (n=108, 95.6%) support formal accreditation of physicians and clinics practicing HLM, indicating widespread professional demand for institutional recognition of this emerging field and underscoring the role of structured education as a catalyst for translating theory into practice.

In addition to educational outcomes, respondents pointed out several systemic barriers impeding the widespread adoption of HLM. One key obstacle was the fee-for-service payment structure, which rewards service volume rather than health outcomes and provides limited incentives for preventive care or time-intensive, individualized lifestyle counseling [17,18]. Another barrier to HLM implementation was the lack of official recognition of HLM by regulatory bodies, national-level regulatory constraints, few accreditation opportunities, and discrepancies in access to diagnostic tools and infrastructure depending on the country of practice. Literature shows that physician accreditation in medical subdomains is correlated with better patient outcomes [19], while national board certification opportunities accompanied by CME education are associated with practice growth, career satisfaction, and better employment opportunities [20]. In this study, 95.6% (n=108) of physicians were in favor of formal accreditation of HLM, and 73.5% (n=83) of physicians expected HLM to significantly grow, eventually integrating into mainstream medicine. The responses represent an urgent need for coordinated efforts that integrate education, policy reform, and infrastructure development to facilitate the full-scale implementation of HLM across health care systems. Therefore, this study identified the need for structured education and certification programs, as well as formal board recognition, to enable scalable, evidence-based delivery of healthy longevity–focused care.

### Limitations

There are several limitations to the study. As with all voluntary survey studies, the findings may be subject to self-selection and nonresponse bias, as physicians with a greater interest in HLM may have been more likely to participate. Outcomes were self-reported and may therefore be influenced by recall or social desirability bias. The cross-sectional design prevents causal inference, limiting conclusions about the long-term impact of HLM education on clinical practice. Selection bias is a concern, as participants were self-selected and may have had a pre-existing interest in HLM, potentially overestimating the adoption of HLM principles. The low response rate also introduces nonresponse bias, as respondents may not be representative of all physicians completing the course. In addition, self-reported data on confidence and practice changes are subject to recall and social desirability biases, lacking objective clinical validation. Regional differences in medical regulations and reimbursement models may further limit generalizability. Practice setting (public vs private) may plausibly influence physicians’ perceptions of the applicability and validity of HLM, given differences in clinical autonomy, reimbursement structures, and preventive care implementation. Despite these limitations, this study provides novel insights into the role of structured online HLM education and underscores the need for further investigation into its clinical integration. Future research should assess the long-term impact and cost-effectiveness of HLM training while evaluating how delivery methods and systemic barriers influence clinical implementation and professional outcomes.

### Conclusions

This study highlights the importance of accredited HLM education as a scalable tool for improving physicians’ competency in HLM. The development and implementation of a designated curriculum constitute a critical step in bridging the translational gap between rapidly advancing theoretical discoveries in geroscience and their application in clinical settings. There is a growing need for structured, competency-based, and frequently updated educational programs that equip health care professionals with the necessary knowledge and skills to integrate gerodiagnostics (HLM diagnostics based on biomarkers of aging) and gerotherapeutics (drugs that affect pathways involved in aging to lessen the burden of age-related diseases as well as to prolong health span and potentially lifespan [21]) into patient care. Importantly, systematic studies of medical education serve as a form of quality control, ensuring the identification of educational gaps and clinical implementation challenges and enabling the iterative optimization of teaching methodologies and content relevance. This process is fundamental for further establishment of standardized training pathways and professional accreditation frameworks, which are essential prerequisites for recognizing HLM as a formal medical discipline. Furthermore, the scalability and long-term sustainability of such educational initiatives require strategic integration with health policy and regulatory efforts, fostering a harmonized approach that supports system-level adoption. Aligning educational standards with policy development not only enhances the credibility of HLM but also ensures that it becomes an integral part of national and international health systems, ultimately advancing the goal of extending health span and reducing the burden of age-related diseases.

## Supplementary material

10.2196/83779 Checklist 1 STROBE checklist.

## Abbreviations

CME: continuing medical education
HLM: healthy longevity medicine
OR: odds ratio
PPG: primary and preventive care and general practice
SPEC: specialized care and advanced procedures
STROBE: Strengthening the Reporting of Observational Studies in Epidemiology
