# Correction: Upskilling in Healthy Longevity Medicine and Its Association With Physicians’ Implementation Intent and Self-Reported Clinical Confidence: Cross-Sectional Observational Study

**DOI:** 10.2196/114811

**Published:** 2026-11-10

**Authors:** Evelyne Bischof, Dominika Wilczok, James L Kirkland, Bhirau Wilaksono, Christine Yuan Huang, Suwanna Suwannaphong, Wanviput Sanphasitvong, Dalila Čamdžić, Carolina Hernandez, Yoko Madea, Hidekazu Yamada, Melissa Alexandre Fernandes, Ricardo Gaminha Pacheco, Fabiano M Serfaty, Fernanda Calvo-Fortes, Amit Goldman, Andrea B Maier, Alexey Moskalev, Morten Scheibye-Knudsen, Alex Zhavoronkov

**Affiliations:** 1 Sheba Longevity Center, Sheba Medical Center, Gray Faculty of Medical & Health Sciences, Tel Aviv University, Sheba Road 2, Ramat Gan, Tel Aviv, 5265601, Israel, 972 0512504301; 2 Shanghai University of Medicine and Health Sciences, Shanghai, China; 3 Duke University, Durham, NC, United States; 4 Duke Kunshan University, Kunshan, Jiangsu, China; 5 Center for Advanced Gerotherapeutics, Division of Endocrinology and Metabolism, Department of Medicine, Cedars Sinai Health Sciences University, Los Angeles, CA, United States; 6 Center for Healthy Longevity, Faculty of Medicine, Universitas Negeri Makassar, Makassar, Indonesia; 7 Department of Biochemistry, Faculty of Medicine, Universitas Negeri Makassar, Makassar, Indonesia; 8 Quantum Life Inc, Hong Kong, China (Hong Kong); 9 VitalLife, Bumrungrad International Hospital, Bangkok, Thailand; 10 Sarajevo School of Science and Technology, Sarajevo, Bosnia and Herzegovina; 11 Departent of Biomedicine, University of Barcelona, Barcelona, Spain; 12 Madea Clinic, Okayama, Japan; 13 Anti-Aging Center, Kindai University, Osaka, Japan; 14 Novartis Institutes for BioMedical Research, Basel, Switzerland; 15 Insilico Medicine Hong Kong Ltd, Hong Kong, China (Hong Kong); 16 Departent of Biomedicine, Universidade do Estado do Rio de Janeiro, Rio de Janeiro, Brazil; 17 School of Nursing, Federal University of Minas Gerais, Belo Horizonte, Brazil; 18 Saïd Business School, University of Oxford, Oxford, United Kingdom; 19 Healthy Longevity Translational Research Program, Yong Loo Lin School of Medicine, National University of Singapore, Singapore, Singapore; 20 NUS Academy for Healthy Longevity, Yong Loo Lin School of Medicine, National University of Singapore, Singapore, Singapore; 21 Department of Human Movement Sciences, Faculty of Behavioral and Movement Sciences, Amsterdam Movement Sciences, Vrije Universiteit Amsterdam, Amsterdam, The Netherlands; 22 Petrovsky Russian Research Center for Surgery, Moscow, Russian Federation; 23 Center for Healthy Aging, Department of Cellular and Molecular Medicine, University of Copenhagen, Copenhagen, Denmark; 24 Insilico Medicine US Inc, Cambridge, MA, United States

In “Upskilling in Healthy Longevity Medicine and Its Association With Physicians’ Implementation Intent and Self-Reported Clinical Confidence: Cross-Sectional Observational Study” [1], the authors have identified the need to provide further details in the Conflicts of Interest section.

The original disclosure identified several authors as volunteers of Longevity Education Hub but did not specify their individual responsibilities in the development, delivery, administration, and promotion of the educational resources evaluated in the study. It also omitted author ABM’s position as chief medical officer of NU. The following correction provides these additional disclosures in the interest of transparency.

In the originally published article, the Conflicts of Interest section read as follows:

*EB, DW, BW, CYH, SS, WS, DČ, CH, YM, HY, MAF, RGP, MS-K, AM, AG, FC-F, FS, and AZ are volunteers of the Longevity Education Hub—the nonprofit online education platform for Healthy Longevity Medicine Courses. AZ is the founder and shareholder of Insilico Medicine, a generative artificial intelligence and quantum computing–enabled longevity biotechnology company. All other authors declare no competing interests. AM is Director of the NUS Academy for Healthy Longevity (disclosure, not a conflict of interest).*

The Conflicts of Interest section has been revised to read as follows:

*EB, DW, BW, CYH, SS, WS, DČ, CH, YM, HY, MAF, RGP, MS-K, AM, AG, FC-F, FMS, and AZ are volunteers of the Longevity Education Hub (LEH), a volunteer-run, noncommercial educational initiative offering free, online Healthy Longevity Medicine courses. None of the authors receives remuneration or holds an ownership interest in LEH. The following authors have additional nonfinancial roles in the initiative and its educational resources evaluated in this study: AZ and EB cofounded the initiative and conceptualized, developed, and authored course curricula and educational materials; MS-K and AM contributed scientific expertise and coauthored educational content; DW and DČ were responsible for communications, social media outreach, website coordination, and operational support, including the dissemination and accessibility of the courses. DČ additionally undertook course management and administrative coordination.*

*AZ is the founder and shareholder of Insilico Medicine, a generative AI and quantum computing–enabled longevity biotechnology company. ABM is chief medical officer of NU, a software company that provides analysis of health data and does not offer educational programs. ABM is also director of the NUS Academy for Healthy Longevity (disclosure, not a conflict of interest). All other authors declare no competing interests.*

This correction clarifies the authors’ disclosed relationships and does not alter the study results, analyses, or conclusions.

The correction will appear in the online version of the paper on the JMIR Publications website together with the publication of this correction notice. Because this was made after submission to PubMed, PubMed Central, and other full-text repositories, the corrected article has also been resubmitted to those repositories.
