# Supplementary material for: Upskilling in Healthy Longevity Medicine and Its Association With Physicians’ Implementation Intent and Self-Reported Clinical Confidence: Cross-Sectional Observational Study
Source: JMIR Med Educ. 2026 Mar 19;12:e83779. doi: 10.2196/83779 (PMC13002000; doi:10.2196/83779)
Supplement: Checklist 1 [file mededu-v12-e83779-s001.docx]

STROBE Statement—checklist of items that should be included in reports of observational studies

|  | Item No. | Recommendation | Page  No. | Relevant text from manuscript |
| --- | --- | --- | --- | --- |
| **Title and abstract** | 1 | (*a*) Indicate the study’s design with a commonly used term in the title or the abstract | 1 | Upskilling in Healthy Longevity Medicine and Its Association With Physicians’ Implementation Intent and Self-Reported Clinical Confidence: Cross-Sectional Observational Study” |
|  |  | (*b*) Provide in the abstract an informative and balanced summary of what was done and what was found | 2 | Background: Structured educational programs for physicians in healthy longevity medicine (HLM) remain scarce. No  published data yet document the impact of longevity-focused medical education on physicians. This study assesses the  ramifications of the HLM curriculum, certified by the American Council for Continuing Medical Education, on physicians’  confidence in their knowledge of HLM and clinical practice.  Objective: This study aimed to evaluate the impact of accredited HLM education on physicians’ confidence in knowledge  and practice patterns, examining self-reported integration of HLM principles, professional attitudes, and career trajectories to  determine the translational value of structured curricula in the emerging medical discipline.  Methods: A cross-sectional online survey was conducted between March and April 2024 among physicians who had  completed accredited HLM courses between January 2023 and February 2024. Invitations were sent globally to 590 eligible  physicians; trainees and students were excluded. A total of 113 (19.2%) respondents completed the survey and were included  in the analysis. The survey assessed self-reported changes in clinical implementation, confidence in HLM-related knowledge,  and professional attitudes following course completion. Descriptive statistics and logistic regression analyses were performed  (P<.05).  Results: Respondents represented 42 nationalities and were primarily trained in family medicine (n=31, 27.4%) and internal  medicine (n=18, 15.9%). Overall, 96.5% (n=99) of the respondents reported increased confidence in HLM-related knowledge,  with 47.8% (n=55) indicating substantial improvement. More than half of the respondents (n=63, 55.8%) reported integrating  HLM principles into routine patient assessments, and 80.5% (n=91) of the respondents reported more frequent discussions  related to health span–focused care. In addition, 23% (n=26) of the respondents initiated aging biomarker testing, 48.7%  (n=55) increased the testing frequency, 52.2% (n=59) reported a shift in their perspective on aging, and 73.5% (n=83)  anticipated full integration of HLM into mainstream medicine. Physicians practicing in specialized care demonstrated higher  odds of reporting increased confidence in HLM knowledge compared with those in primary and preventive care (odds ratio  4.46, 95% CI 1.55‐12.79; P=.005).  Conclusions: Accredited education in HLM is associated with enhanced confidence in HLM knowledge, increased clinical  engagement with HLM practices, and a shift in aging-related care paradigms. These findings underscore the critical role of  structured HLM curricula in bridging the translational gap between geroscience and everyday medical practice. Nevertheless,  systemic health care barriers impede widespread implementation, warranting policy-level strategies to support health span–  oriented education and care models. |
| Introduction | | | |  |
| Background/rationale | 2 | Explain the scientific background and rationale for the investigation being reported | 2 | Structured educational programs for physicians in healthy longevity medicine (HLM) remain scarce. No  published data yet document the impact of longevity-focused medical education on physicians. This study assesses the ramifications of the HLM curriculum, certified by the American Council for Continuing Medical Education, on physicians’  confidence in their knowledge of HLM and clinical practice |
| Objectives | 3 | State specific objectives, including any prespecified hypotheses | 2 | This study aimed to evaluate the impact of accredited HLM education on physicians’ confidence in knowledge  and practice patterns, examining self-reported integration of HLM principles, professional attitudes, and career trajectories to  determine the translational value of structured curricula in the emerging medical discipline. |
| Methods | | | |  |
| Study design | 4 | Present key elements of study design early in the paper | 3 | This cross-sectional observational study was conducted  between March and April 2024. Physicians who completed  the Longevity Education Hub’s online Longevity Medicine  101 and/or Longevity Medicine 201 courses self-adminis-  tered and remotely completed the anonymous online survey  assessing changes in clinical practice, confidence in HLM  knowledge, and perspectives on HLM |
| Setting | 5 | Describe the setting, locations, and relevant dates, including periods of recruitment, exposure, follow-up, and data collection | 3 | The study was conducted between March and April 2024 using a global, anonymous online survey distributed via Google Forms |
| Participants | 6 | (*a*) *Cohort study*—Give the eligibility criteria, and the sources and methods of selection of participants. Describe methods of follow-up  *Case-control study*—Give the eligibility criteria, and the sources and methods of case ascertainment and control selection. Give the rationale for the choice of cases and controls  *Cross-sectional study*—Give the eligibility criteria, and the sources and methods of selection of participants | 3 | Eligibility criteria included physicians who completed the "Longevity Medicine 101" or "201" courses via Teachable between December 2021 and March 2024. Exclusion criteria removed non-MDs, those who did not pass final quizzes, and junior MDs/students to focus on practicing physicians |
|  |  | (*b*) *Cohort study*—For matched studies, give matching criteria and number of exposed and unexposed  *Case-control study*—For matched studies, give matching criteria and the number of controls per case | - | - |
| Variables | 7 | Clearly define all outcomes, exposures, predictors, potential confounders, and effect modifiers. Give diagnostic criteria, if applicable | 6 | Primary outcomes included self-reported confidence in HLM-related knowledge (Likert scale) and integration of HLM principles into routine clinical assessments (frequency-based responses). Secondary outcomes included changes in frequency of longevity-related patient conversations, initiation or increased use of aging biomarker testing, perceived impact of HLM education on demographic challenges, shifts in perspective on aging and healthy longevity, expectations for the future growth and formalisation of HLM, and career impacts (e.g., opening or joining HLM clinics). The main explanatory variable in regression analyses was specialty group, categorized as Primary and Preventive Care & General Practice (PPG) versus Specialized Care & Advanced Procedures (SPEC).. |
| Data sources/ measurement | 8* | For each variable of interest, give sources of data and details of methods of assessment (measurement). Describe comparability of assessment methods if there is more than one group | *6* | Data was sourced from a 20-item survey instrument validated by experts and pilot-tested on 20 physicians, showing high internal consistency (Cronbach's alpha of 0.72) |
| Bias | 9 | Describe any efforts to address potential sources of bias | 8 | Potential sources of bias mentioned include self-selection and non-response bias (interested physicians more likely to respond), as well as recall and social desirability bias due to the self-reported nature of the data . |
| Study size | 10 | Explain how the study size was arrived at | 3-4 | The study invited **all 590 eligible practicing physicians** who met the inclusion criteria after systematic exclusions from the original 6,464 course enrollees; **113 physicians completed the survey and were included in the analysis**. No a priori sample size or power calculation was performed; instead, all eligible physicians were approached, and the final sample size was determined by the response rate. |

Continued on next page

| Quantitative variables | 11 | Explain how quantitative variables were handled in the analyses. If applicable, describe which groupings were chosen and why | 6 | Quantitative variables were collected as ordered categorical responses (Likert scales for confidence and perspectives; frequency categories for clinical behaviors). For descriptive analyses, these categories were summarized as counts and percentages. For logistic regression, outcomes were dichotomised (e.g., reporting increased confidence vs. not), and the main predictor was specialty group (SPEC vs. PPG, with PPG as the reference). However, the manuscript does **not** explicitly report the exact cut-points used to collapse each Likert or frequency scale into binary outcomes. |
| --- | --- | --- | --- | --- |
| Statistical methods | 12 | (*a*) Describe all statistical methods, including those used to control for confounding | 6 | Statistical analyses were conducted using R (v4.2.0). Descriptive statistics, including counts and percentages, were used to summarize demographics and overall survey responses. To assess between-group differences (Specialized Care vs. Primary and Preventive Care) in course-related outcomes, separate logistic regression models were employed for each outcome. These models utilized the Primary and Preventive Care group as the reference category to control for practice-type variables. |
|  |  | (*b*) Describe any methods used to examine subgroups and interactions | 4 | Subgroups and interactions: The study examined subgroups by categorizing medical specialties into two distinct roles: Primary and Preventive Care & General Practice (PPG) and Specialized Care & Advanced Procedures (SPEC). Logistic regression analysis was then used to identify differences in educational takeaways and clinical behaviors between these two groups. |
|  |  | (*c*) Explain how missing data were addressed | 6 | Because the Google Forms survey platform required participants to complete all essential items before submission, there were no missing values for mandatory variables. Consequently, no data imputation or casewise exclusion was necessary. |
|  |  | (*d*) *Cohort study*—If applicable, explain how loss to follow-up was addressed  *Case-control study*—If applicable, explain how matching of cases and controls was addressed  *Cross-sectional study*—If applicable, describe analytical methods taking account of sampling strategy | - | Sampling methods not applicable |
|  |  | (*e*) Describe any sensitivity analyses | - | Sensitivity analyses were not performed |
| Results | | | | |
| Participants | 13* | (a) Report numbers of individuals at each stage of study—eg numbers potentially eligible, examined for eligibility, confirmed eligible, included in the study, completing follow-up, and analysed | 3 | the study details the progression from 6,464 enrolled students to 3,223 course completers, then to 1,662 MDs, 590 eligible practicing physicians, and finally 113 survey respondents. |
|  |  | (b) Give reasons for non-participation at each stage | 3 | Reasons for exclusion included failure to complete modules (3,241), lack of MD title (1,561), and being a junior MD or student (1,072) |
|  |  | (c) Consider use of a flow diagram | 4 | Diagram was applied and is reported as figure 1 of the paper. |
| Descriptive data | 14* | (a) Give characteristics of study participants (eg demographic, clinical, social) and information on exposures and potential confounders | 4 | The study provides participant characteristics including nationality (representing 42 countries), medical specialty, and practice setting (public vs. private) . Primary specialties identified were family medicine (27.4%) and internal medicine (25.6%). |
|  |  | (b) Indicate number of participants with missing data for each variable of interest | 6 | No missing data were recorded |
|  |  | (c) *Cohort study*—Summarise follow-up time (eg, average and total amount) |  |  |
| Outcome data | 15* | *Cohort study*—Report numbers of outcome events or summary measures over time |  |  |
|  |  | *Case-control study—*Report numbers in each exposure category, or summary measures of exposure |  |  |
|  |  | *Cross-sectional study—*Report numbers of outcome events or summary measures | *6-7* | For each outcome, the manuscript reports the number and proportion of respondents in each category. For example, 99/113 (96.5%) reported increased confidence in HLM knowledge (with 54/113, 47.8%, indicating substantial improvement); 63/113 (55.8%) integrated HLM principles into routine patient assessments; 91/113 (80.5%) reported more frequent healthy longevity discussions with patients; 26/113 (23%) initiated aging biomarker testing; 55/113 (48.7%) increased the frequency of such testing; 59/113 (52.2%) reported a paradigm shift in their perception of aging; and 83/113 (73.5%) anticipated full integration of HLM into mainstream medical practice. |
| Main results | 16 | (*a*) Give unadjusted estimates and, if applicable, confounder-adjusted estimates and their precision (eg, 95% confidence interval). Make clear which confounders were adjusted for and why they were included | 6 | The outcomes were originally measured using ordered categories (Likert and frequency scales), and these were subsequently dichotomised for logistic regression (e.g., “increased confidence” vs. “no increase”). However, the manuscript does **not** specify the exact category boundaries used for each dichotomy (i.e., which response options were grouped together), so these boundaries are not explicitly reported. |
|  |  | (*b*) Report category boundaries when continuous variables were categorized | - | There were no category boundaries |
|  |  | (*c*) If relevant, consider translating estimates of relative risk into absolute risk for a meaningful time period | - | Risk estimates are not applicable |

Continued on next page

| Other analyses | 17 | Report other analyses done—eg analyses of subgroups and interactions, and sensitivity analyses | 7 | Beyond the primary descriptive results and main logistic regression models, the study reports additional analyses of career-related outcomes (e.g., opening or joining HLM clinics, joining HLM physician groups) in Figure 3, and compares multiple educational and behavioral outcomes between PPG and SPEC in Figure 4. No sensitivity analyses or interaction tests were conducted. |
| --- | --- | --- | --- | --- |
| Discussion | | | | |
| Key results | 18 | Summarise key results with reference to study objectives | 7 | This study found that accredited, online HLM courses are  associated with significant improvements in physician-repor-  ted confidence in the knowledge of HLM and increased  clinical integration of healthy longevity–focused practices. |
| Limitations | 19 | Discuss limitations of the study, taking into account sources of potential bias or imprecision. Discuss both direction and magnitude of any potential bias | 8 | The cross-sectional design prevents causal inference; self-selection and non-response bias may overestimate adoption; and the reliance on self-reported data introduces potential recall or social desirability bias . Regional differences in regulations and reimbursement also impact the results |
| Interpretation | 20 | Give a cautious overall interpretation of results considering objectives, limitations, multiplicity of analyses, results from similar studies, and other relevant evidence | 8 | The results are interpreted as a suggestion that structured curricula can bridge the translational gap between geroscience and medical practice. |
| Generalisability | 21 | Discuss the generalisability (external validity) of the study results | 8 | The study acknowledges that generalisability may be limited by regional medical regulations, reimbursement models, and the specific practice settings (public vs. private) of the participants |
| Other information | |  | | |
| Funding | 22 | Give the source of funding and the role of the funders for the present study and, if applicable, for the original study on which the present article is based | 9 | No funding was obtained for this study |

*Give information separately for cases and controls in case-control studies and, if applicable, for exposed and unexposed groups in cohort and cross-sectional studies.

**Note:** An Explanation and Elaboration article discusses each checklist item and gives methodological background and published examples of transparent reporting. The STROBE checklist is best used in conjunction with this article (freely available on the Web sites of PLoS Medicine at http://www.plosmedicine.org/, Annals of Internal Medicine at http://www.annals.org/, and Epidemiology at http://www.epidem.com/). Information on the STROBE Initiative is available at www.strobe-statement.org.
